# Supplementary material for: Electron-flux infrared response to varying π-bond topology in charged aromatic monomers
Source: Nat Commun. 2016 Aug 31;7:12633. doi: 10.1038/ncomms12633 (PMC5013661; doi:10.1038/ncomms12633)
Supplement: Supplementary Information — Supplementary Figures 1-4, Supplementary Tables 1-23, Supplementary Note 1 and Supplementary Reference [file ncomms12633-s1.pdf]

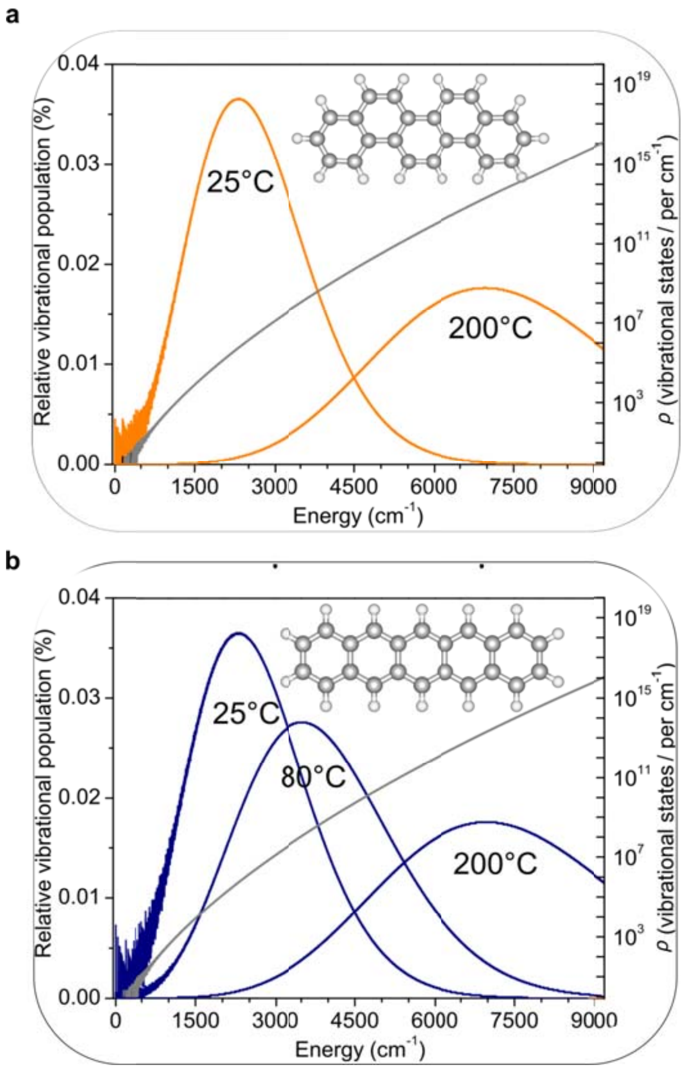

Supplementary Figure 1 | Vibrational ion distributions and density of vibrational states (per cm^{-1}). a, picene$^+$, **b**, pentacene$^+$. Thermal ion fraction distributions of picene and pentacene cations calculated at 20, 80 and 200 °C. To calculate the density of vibrational states ρ we employed the direct counting method based on the Beyer-Swinehart algorithm and the spin-doublet ground-state mode frequencies of picene$^+$ and pentacene$^+$. We used a logarithmic scale to plot ρ.

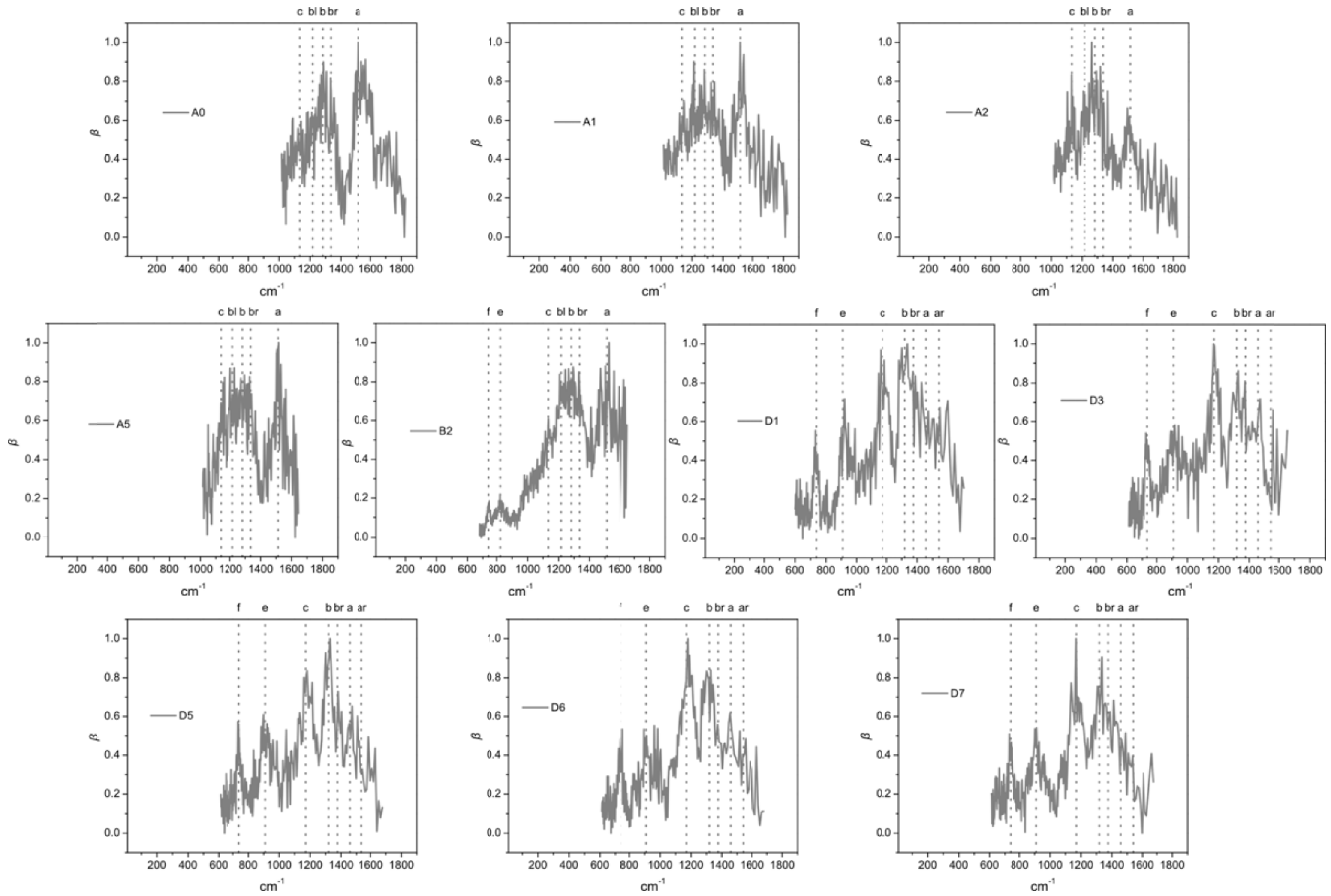

Supplementary Figure 2 | Data sample of dissociation yield *β* functions retrieved from spectral scans (single FEL-ion-trap measurements) of multiple-photon product ions. The spectral scans A0, A1, A2, A5 and B2 correspond to picene$^+$, and the spectral scans D1, D3, D5, D6 and D7 correspond to pentacene$^+$. The dotted lines show the final frequency peak positions of measured bands (Fig. 3, Table 1). These typical single measurements exhibit large statistical random fluctuations taking place during experiments but which cancel out upon the averaging procedure (see for instance the partial spectra averages in Supplementary Fig. 3,4).

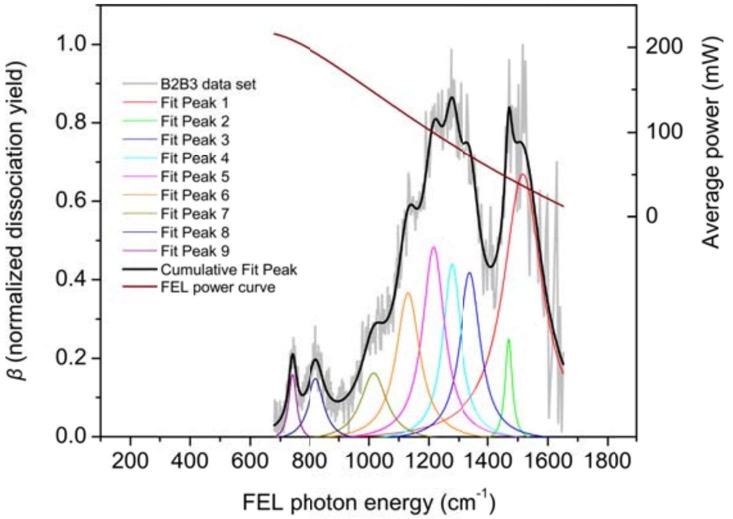

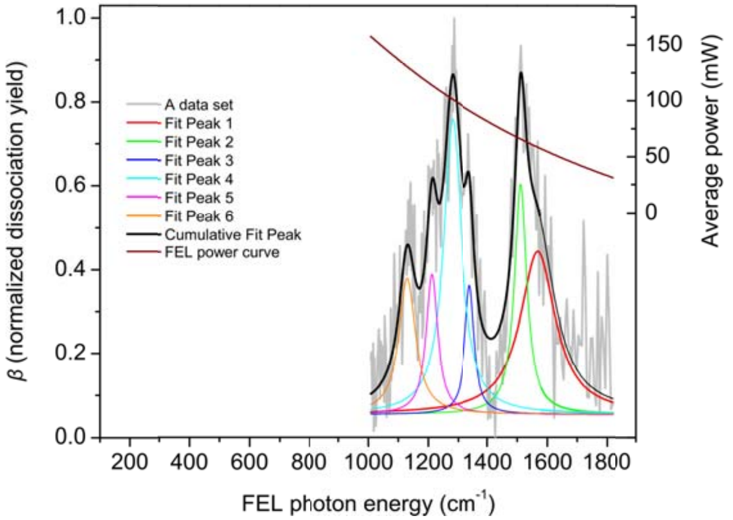

Supplementary Figure 3 | Normalized partial action spectra (*β*) of picene$^+$ corrected from FEL power variations. (**top**) IR action spectrum retrieved from a data set sample (B2B3) composed of 2 FEL spectral scans of parent and product ions. (**bottom**) IR action spectrum retrieved from a data set sample (A) composed of 5 FEL spectral scans of parent and product ions. Both data sets were measured within a week apart in independent FEL-ion-trap experimental sessions. Apart from the difference in band broadenings and resolution, the spectral deconvolution band fits (Supplementary Tables 3, 4) reveal that spectrum B2B3 reproduces the absorption features seen in spectrum A in the 1000-1650 cm^{-1} range. Data set labels are arbitrary.

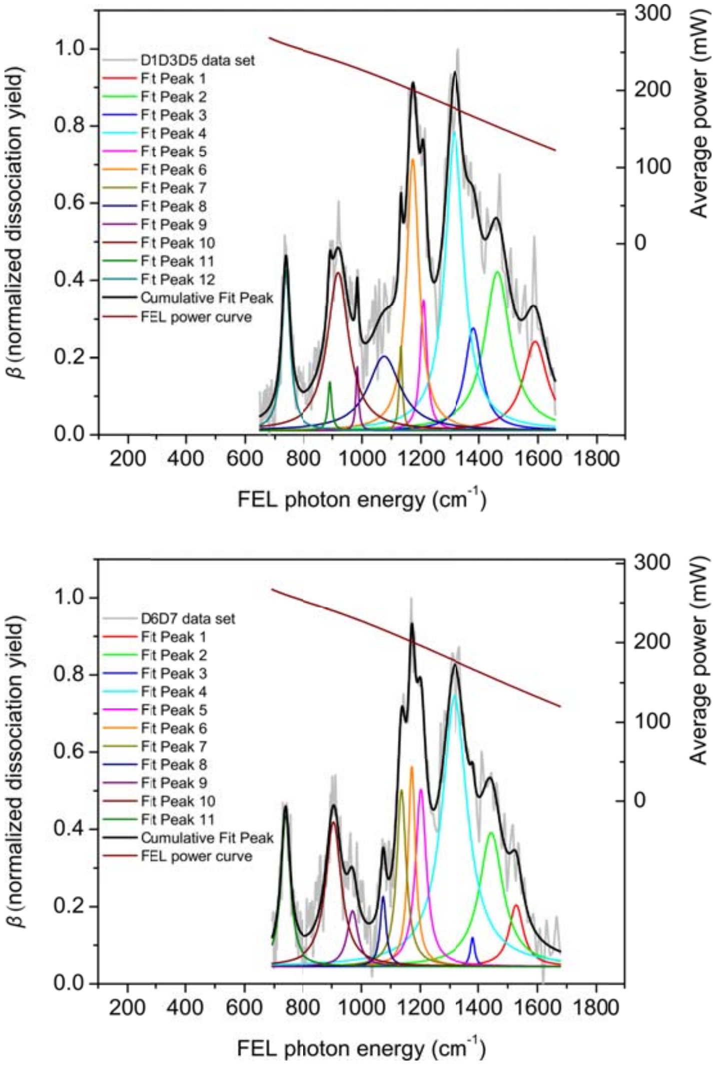

Supplementary Figure 4 | Normalized partial action spectra (*β*) of pentacene$^+$ corrected from FEL power variations. (**top**) IR action spectrum retrieved from a data set sample (D1D3D5) composed of 4 FEL spectral scans of parent and product ions. (**bottom**) IR action spectrum retrieved from a data set sample (D6D7) composed of 2 FEL spectral scans of parent and product ions. Both data sets were measured during a FEL-ion-trap experimental session. An additional data sample set of 6 FEL scans (not shown) was recorded within a week apart. The spectral deconvolution band fits (Supplementary Tables 5, 6) applied to partial spectra retrieved from all data sets show the absorption features exhibited in the final spectrum. Data set labels are arbitrary.

Supplementary Table 1 | Electronic configurations and π states of picene

One-electron excitation	State (eV)	Electronic single configuration
neutral	$^1A_1$	$(1a_1)^2(2a_1)^2(1b_1)^2(1a_2)^2(1b_2)^2(2a_2)^2(2b_1)^2(3b_1)^2(3a_2)^2(4a_2)^2(4b_1)^2$
charged<br>*radical cation*	$^2B_1$<br>(0.0)	$(1a_1)^2(1b_1)^1(2a_1)^1(2a_1)^1(1b_2)^1(1b_2)^1(1b_1)^1(1a_2)^2(2a_2)^2(2b_1)^2(3b_1)^2$<br>$(3a_2)^2(4a_2)^2(4b_1)^1(4b_1)^0$
$4a_2 \rightarrow 4b_1$<br>(↓)	$^2A_2$<br>(0.28)	$(1a_1)^2(1a_1)^2(2b_1)^2(2a_1)^1(1b_2)^1(1b_2)^1(1a_2)^1(1a_2)^1(2b_1)^2(3b_1)^2$<br>$(3a_2)^2(4a_2)^1(4b_1)^1(4b_1)^1(4a_2)^0$
$4b_1 \rightarrow 5a_2$(*)<br>(↑)	$^2A_2$<br>(2.66)	$(1a_1)^2(1b_2)^2(1b_1)^1(2a_1)^1(2a_1)^1(1a_2)^1(1a_2)^1(1b_1)^1(2a_2)^2(2b_1)^2(3b_1)^2$<br>$(3a_2)^2(4a_2)^2(5a_2)^1(4b_1)^0$
$4a_2 \rightarrow 5b_1$<br>(↑)	$^2A_2$<br>(3.56)	$(1a_1)^2(2a_1)^2(1b_1)^2(1a_2)^1(1b_2)^1(1b_2)^1(1a_2)^1(2a_2)^2(2b_1)^2(3b_1)^2$<br>$(3a_2)^2(4b_1)^1(4a_2)^1(5b_1)^1(4b_1)^0$
$3b_1 \rightarrow 5a_2$(*)<br>(↓)	$^2A_2$<br>(4.32)	$(1a_1)^2(2a_1)^2(1b_2)^2(1a_2)^2(1b_1)^2(2a_2)^2(2b_1)^2(3b_1)^1$<br>$(3a_2)^1(3a_2)^1(4a_2)^1(4a_2)^1(5a_2)^1(4b_1)^1(3b_1)^0$

(*) Unbound excited states. Vertical arrows represent α (↑) and β (↓) electrons.

Supplementary Table 2 | Electronic configurations and π states of pentacene

One-electron excitation	State (eV)	Electronic single configuration
neutral	^1A_g	$(1b_{3g})^2(1b_{2u})^2(1a_g)^2(1b_{1g})^2(1b_{2g})^2(1a_u)^2(1b_{3u})^2(2b_{2g})^2(2b_{1g})^2(2a_u)^2(3b_{2g})^2$
charged<br>*radical cation*	$^2B_{2g}$<br>(0.0)	$(1b_{3g})^2(1b_{2u})^2(1a_g)^2(1b_{1g})^2(1b_{2g})^2(1a_u)^2(1b_{3u})^2(2b_{2g})^2(2b_{1g})^2(2a_u)^2(3b_{2g})^1$<br>$(3b_{2g})^0$
$3b_{2g} \rightarrow 2b_{3u}$<br>(↑)	$^2B_{3u}$<br>(1.18)	$(1b_{3g})^2(1b_{2u})^2(1a_g)^2(1b_{1g})^2(1b_{2g})^2(1a_u)^2(1b_{3u})^2(2b_{2g})^2(2b_{1g})^2$<br>$(2a_u)^2(2b_{3u})^1(3b_{2g})^0$
$2a_u \rightarrow 3b_{2g}$<br>(↓)	^2A_u<br>(1.27)	$(1b_{3g})^2(1b_{2u})^2(1a_g)^2(1b_{1g})^2(1b_{2g})^2(1a_u)^2(1b_{3u})^2(2b_{2g})^2(2b_{1g})^2$<br>$(2a_u)^1(3b_{2g})^1(3b_{2g})^1(2a_u)^0$
$2b_{1g} \rightarrow 2b_{3u}$<br>(↑)	^2A_u<br>(2.70)	$(1b_{3g})^2(1b_{2u})^2(1a_g)^2(1b_{2g})^1(1b_{1g})^1(1b_{1g})^1(1b_{2g})^1(1a_u)^2(1b_{3u})^2(2b_{2g})^1(2b_{1g})^1$<br>$(2a_u)^1(2b_{2g})^1(3b_{2g})^1(2a_u)^1(2b_{3u})^1(3b_{2g})^0$
$2b_{1g} \rightarrow 2b_{3u}$<br>(↓)	^2A_u<br>(3.23)	$(1b_{3g})^2(1b_{2u})^2(1a_g)^2(1a_g)^1(1b_{1g})^1(1b_{2g})^2(1a_u)^2(1b_{3u})^2(2b_{2g})^2(2b_{1g})^1$<br>$(2a_u)^1(2a_u)^1(2b_{3u})^1 (3b_{2g})^1(2b_{1g})^0$

Vertical arrows represent α (↑) and β (↓) electrons.

Supplementary Table 3 | Characterization of dissociation yield (*β*) action bands of picene$^+$ (B2B3 data set) normalized and corrected from FEL power curve

IR multiple-photon action			Deconvoluted band fit components							
band	$\tilde{\nu}_{exp}$	*β*	*Peak*	$\tilde{\nu}_c$	*ε*	*w*	*ε*	*A*	*ε*	*h*
a_r	1512	0.7509	1	1517	*6.56*	157	*12.92*	168	*21.07*	0.68
a	1472	0.8402	2	1470	*2.31*	29	*12.75*	12	*6.96*	0.26
b_r	1335	0.7475	3	1340	*7.13*	87	*21.89*	59	*26.72*	0.43
b	1278	0.8651	4	1277	*5.40*	76	*33.87*	54	*44.45*	0.45
b_l	1222	0.8111	5	1216	*6.51*	90	*31.10*	70	*42.11*	0.50
c	1136	0.5902	6	1131	*5.38*	97	*22.38*	58	*20.94*	0.38
d	1025	0.2897	7	1016	*7.01*	106	*25.55*	29	*9.45*	0.17
e	823	0.1967	8	822	*3.29*	68	*13.74*	17	*3.79*	0.16
f	740	0.2103	9	740	*1.86*	32	*7.40*	9	*2.05*	0.17

Definition of column headings in Table 2. Statistics: Reduced Chi-Sqr 3.97E-03, Residual Sum of Squares 1.65, R-Square (COD) 0.9467, Adj. R-Square 0.94323.

Supplementary Table 4 | Characterization of dissociation yield (*β*) action bands of picene$^+$ (A data set) normalized and corrected from FEL power curve

IR multiple-photon action			Deconvoluted band fit components							
band	$\tilde{\nu}_{exp}$	*β*	*Peak*	$\tilde{\nu}_c$	*ε*	*w*	*ε*	*A*	*ε*	*h*
a_r	1573	0.538	1	1569	*19.45*	142	*26.14*	87	*30.11*	0.39
a	1513	0.871	2	1512	*3.12*	50	*16.52*	43	*22.16*	0.55
b_r	1337	0.6355	3	1341	*4.17*	38	*17.00*	19	*10.45*	0.31
b	1281	0.8666	4	1282	*3.25*	74	*17.24*	82	*21.41*	0.70
b_l	1214	0.6203	5	1212	*4.04*	47	*17.22*	25	*10.96*	0.33
c	1130	0.4602	6	1130	*4.17*	67	*13.08*	34	*5.96*	0.33

Definition of column headings in Table 2. Statistics: Reduced Chi-Sqr 0.01157, Residual Sum of Squares 2.35982, R-Square(COD) 0.80286, Adj. R-Square 0.78643.

Supplementary Table 5 | Characterization of dissociation yield (*β*) action bands of pentacene$^+$ (D1D3D5 data set) normalized and corrected from FEL power curve

IR multiple-photon action			Deconvoluted band fit components							
band	$\tilde{\nu}_{exp}$	β	*Peak*	$\tilde{\nu}_c$	ε	*w*	ε	*A*	ε	*h*
a_r	1584	0.3354	1	1591	*3.98*	99	*16.17*	36	*6.36*	0.23
a	1462	0.5618	2	1463	*3.32*	104	*14.53*	68	*12.04*	0.41
b_r	1374	0.65253	3	1382	*3.47*	68	*19.01*	29	*11.64*	0.27
b	1316	0.9412	4	1315	*1.48*	80	*4.30*	97	*7.54*	0.77
c_r	1207	0.76655	5	1209	*1.12*	27	*4.58*	14	*3.40*	0.34
c	1172	0.91211	6	1172	*1.03*	52	*4.97*	57	*6.66*	0.70
c_l	1133	0.6241	7	1132	*0.83*	13	*3.40*	4	*1.25*	0.22
d	1071	0.31694	8	1076	*5.54*	132	*24.53*	40	*8.05*	0.19
e_r	984	0.40702	9	984	*0.83*	11	*2.87*	3	*0.65*	0.17
e	918	0.48598	10	920	*1.81*	88	4.58	56	3.85	0.41
e_l	893	0.4790	11	892	*1.23*	15	5.41	3	1.21	0.13
f	737	0.465468	12	737	*0.44*	33	1.71	22	1.06	0.41

Definition of column headings in Table 2. Statistics: Reduced Chi-Sqr 2.56E-03, Residual Sum of Squares 2.11, R-Square (COD) 0.95008, Adj. R-Square 0.9479.

Supplementary Table 6 | Characterization of dissociation yield (*β*) action bands of pentacene$^+$ (D6D7 data set) normalized and corrected from FEL power curve

IR multiple-photon action			Deconvoluted band fit components							
band	$\tilde{\nu}_{exp}$	β	*Peak*	$\tilde{\nu}_c$	ε	*w*	ε	*A*	ε	*h*
a_r	1524	0.3472	1	1529	*4.10*	58	*16.13*	15	*5.09*	0.16
a	1441	0.534	2	1444	*2.81*	97	*15.81*	53	*9.57*	0.35
b_r	1379	0.5751	3	1381	*3.50*	18	*14.50*	2	*1.83*	0.08
b	1319	0.8281	4	1318	*1.33*	109	*6.08*	121	*7.33*	0.70
c_r	1199	0.7951	5	1202	*1.87*	44	*5.40*	32	*5.71*	0.46
c	1172	0.9338	6	1171	*0.94*	30	*4.88*	24	*5.78*	0.52
c_l	1139	0.7198	7	1136	*1.18*	39	*3.76*	28	*3.34*	0.46
d	1075	0.3536	8	1074	*1.23*	24	*4.23*	7	*1.06*	0.18
e_r	967	0.304	9	971	*2.23*	44	*7.83*	10	*1.77*	0.14
e	906	0.4632	10	906	1.10	64	3.98	38	2.58	0.38
e_l	-	-	-	-	-	-	-	-	-	-
f	739	0.4601	11	738	0.49	38	1.98	23	1.26	0.39

Definition of column headings in Table 2. Statistics: Reduced Chi-Sqr 2.61E-03, Residual Sum of Squares 2.04, R-Square (COD) 0.94374, Adj. R-Square 0.94137.

Supplementary Tables 7—16 | Calculated harmonic frequencies (freq, cm^{-1}) and IR activity (INT, km/mol) of vibrational normal modes (*ip* = in-plane, *oop* = out-of-plane) of pentacene and picene in neutral (spin-singlet) and charged (spin-doublet) electronic states:

Supplementary Table 7 | PENTACENE (B3LYP/6-311G**)

mode	0.97*freq	INT	SYM
102	37.3	0.4132	B3U
59	69.1	0	AU
25	99.21	0	B1G
76	115.67	1.0398	B1U
34	144.6	0	B2G
101	187.47	1.1754	B3U
51	231.26	0	B3G
58	233.57	0	AU
18	255.68	0	AG
24	285.02	0	B1G
33	338.9	0	B2G
75	351.29	0.1492	B1U
100	368.86	0.0119	B3U
50	440.68	0	B3G
99	457.46	35.0973	B3U
23	458.84	0	B1G
98	464.11	3.7714	B3U
22	465.54	0	B1G
93	476.27	9.0702	B2U
49	497.55	0	B3G
57	509.89	0	AU
32	537.02	0	B2G
74	562.06	2.9548	B1U
17	598.09	0	AG
92	620.31	5.2743	B2U
16	627.87	0	AG
56	694.33	0	AU
48	703.49	0	B3G
91	722.24	3.6652	B2U
97	724.27	78.0234	B3U
21	729.7	0	B1G
31	730.42	0	B2G
15	739.41	0	AG
55	748.27	0	AU
30	751.88	0	B2G
14	771.75	0	AG
73	812.56	0.1291	B1U
29	817.57	0	B2G
96	817.58	16.9643	B3U
54	823.36	0	AU
28	850.09	0	B2G
90	862.77	0.2207	B2U

20	863.63	0	B1G
53	875.74	0	AU
27	890.27	0	B2G
72	894.55	2.3087	B1U
95	895.86	87.7544	B3U
47	908.36	0	B3G
19	944.16	0	B1G
94	944.62	5.8256	B3U
52	965.74	0	AU
26	965.77	0	B2G
89	985.92	8.0178	B2U
13	988.59	0	AG
88	1100.06	1.2293	B2U
71	1109.88	7.5758	B1U
46	1123.8	0	B3G
12	1149.3	0	AG
87	1152.76	0.1773	B2U
45	1163.96	0	B3G
11	1170.93	0	AG
70	1173.86	2.6941	B1U
44	1205.07	0	B3G
86	1213.6	1.7052	B2U
69	1255.54	0.0011	B1U
43	1256.35	0	B3G
42	1273.02	0	B3G
68	1279.45	21.5314	B1U
10	1293.73	0	AG
85	1318.05	12.6057	B2U
67	1335.22	2.8714	B1U
9	1368.72	0	AG
84	1375.09	0.7279	B2U
41	1376.6	0	B3G
83	1384.37	4.596	B2U
8	1391.6	0	AG
82	1433.03	0.1024	B2U
66	1434.14	0.7441	B1U
7	1448.45	0	AG
81	1496.27	2.6071	B2U
6	1511.38	0	AG
5	1529.45	0	AG
80	1531.61	2.1647	B2U
40	1546.38	0	B3G
39	1584.63	0	B3G
65	1592.92	0.4221	B1U
38	1622.99	0	B3G
64	1623.8	10.7407	B1U

63	3060.68	2.6937	B1U
37	3061.88	0	B3G
4	3062.37	0	AG
62	3063.79	0.2068	B1U
79	3063.94	3.7659	B2U
36	3064.73	0	B3G
61	3066.39	51.0024	B1U
3	3067.23	0	AG
78	3069.14	4.1779	B2U
2	3070.15	0	AG
35	3081.57	0	B3G
60	3081.6	70.4011	B1U
77	3093.04	94.0542	B2U
1	3093.22	0	AG
	**TOTAL INT**	560.665	

	**Total INT AU**	0	0	%
ip	**Total INT B1U**	177.46	31.65	%
ip	**Total INT B2U**	154.16	27.49	%
oop	**Total INT B3U**	229.03	40.85	%
	**Total INT**	560.67	100	%

**INT B3U / (INT B2U + INT B1U)**	0.690643827
**INT B3U / INT B2U**	1.485691656
**INT CHstr /INT CHoop**	0.988059129

Supplementary Table 8 | PENTACENE$^+$ (B3LYP/6-311G)**

mode	0.97*freq	INT	SYM
102	36.58	0.6351	B3U
59	68.94	0	AU
25	96.1	0	B1G
76	114.68	0.7847	B1U
34	145.28	0	B2G
101	180.94	1.8319	B3U
51	231.68	0	B3G
58	236.05	0	AU
18	254.81	0	AG
24	275.21	0	B1G
33	347	0	B2G
75	352.59	0.1409	B1U
100	374.9	0.0367	B3U
99	434.58	37.0952	B3U
50	435.28	0	B3G
23	444.71	0	B1G
22	458.02	0	B1G
98	468.5	10.6607	B3U
93	474.15	0.2143	B2U
49	490.28	0	B3G
57	497.45	0	AU
32	526.7	0	B2G
74	563.44	2.0221	B1U
17	595.32	0	AG
92	616.53	0.4946	B2U
16	618.47	0	AG
56	680.34	0	AU
48	706.45	0	B3G
31	719.42	0	B2G
91	729.64	2.7005	B2U
15	739.25	0	AG
55	743.27	0	AU
97	744.37	91.2653	B3U
21	745.29	0	B1G
30	745.51	0	B2G
14	779.22	0	AG
73	818.38	0.0704	B1U
29	838.69	0	B2G
54	847.61	0	AU
96	854.5	13.9011	B3U
90	869.32	0.0795	B2U
28	872.69	0	B2G

72	891.79	1.3023	B1U
20	893.25	0	B1G
53	897.85	0	AU
47	907.25	0	B3G
27	917.58	0	B2G
95	926.33	63.7168	B3U
19	965.03	0	B1G
94	965.63	8.1831	B3U
52	993.23	0	AU
26	993.27	0	B2G
89	1006.92	0.484	B2U
13	1007.99	0	AG
71	1112.99	3.6252	B1U
46	1124.13	0	B3G
88	1141	64.635	B2U
12	1160.59	0	AG
45	1161.26	0	B3G
87	1164.24	185.138	B2U
70	1178.02	1.3487	B1U
11	1181.9	0	AG
86	1221.48	170.1685	B2U
44	1222.83	0	B3G
69	1261.66	2.0119	B1U
43	1262.26	0	B3G
42	1272.03	0	B3G
68	1284.89	35.9313	B1U
10	1289.06	0	AG
67	1330.15	7.9995	B1U
85	1355.35	683.7897	B2U
9	1368.5	0	AG
84	1372.98	342.8514	B2U
8	1383.97	0	AG
83	1384.3	264.9197	B2U
41	1389.49	0	B3G
82	1433.96	17.8981	B2U
66	1437.71	5.2389	B1U
7	1459.62	0	AG
81	1481.52	417.581	B2U
6	1502.6	0	AG
40	1509.34	0	B3G
80	1520.73	262.9024	B2U
39	1520.82	0	B3G
5	1530.38	0	AG
65	1569.24	0.318	B1U
64	1588.97	25.813	B1U
38	1598.67	0	B3G

63	3078.88	0.3228	B1U
37	3080.47	0	B3G
4	3080.73	0	AG
79	3082.14	0.8652	B2U
62	3082.64	2.1301	B1U
3	3084.47	0	AG
61	3085.23	0.9725	B1U
36	3085.25	0	B3G
78	3088.81	2.6005	B2U
2	3088.97	0	AG
35	3099.46	0	B3G
60	3099.47	9.7056	B1U
77	3108.94	18.9938	B2U
1	3109	0	AG
	**TOTAL INT**	2763.38	

	**Total INT AU**	0	0	%
ip	**Total INT B1U**	99.7379	3.61	%
ip	**Total INT B2U**	2436.3162	88.16	%
oop	**Total INT B3U**	227.3259	8.22	%
	**Total INT**	2763.38	100	%

**INT B3U / (INT B2U + INT B1U)**	0.089637638
**INT B3U / INT B2U**	0.093307223
**INT CHstr /INT CHoop**	0.156561571

Supplementary Table 9 | PENTACENE$^+$ (ROHF/3-611G**)

mode	0.97*freq	INT	SYM
102	37.09	0.3195	B3U
59	75.68	0	AU
25	103.34	0	B1G
76	109.61	9.0772	B1U
34	159.23	0	B2G
101	196.44	2.5358	B3U
51	247.04	0	B3G
58	256.95	0	AU
18	269.13	0	AG
24	297.33	0	B1G
75	368.05	8.0929	B1U
33	379.32	0	B2G
100	409.17	0.0045	B3U
50	457.67	0	B3G
99	465.35	47.1646	B3U
23	481.78	0	B1G
93	501.32	4.8145	B2U
22	503.44	0	B1G
98	514.64	15.9407	B3U
49	517.32	0	B3G
57	535.25	0	AU
32	564.9	0	B2G
74	573.94	71.9203	B1U
17	629.36	0	AG
92	654.4	0.599	B2U
16	655.02	0	AG
56	733.29	0	AU
48	748.52	0	B3G
31	769.41	0	B2G
91	774.14	0.3487	B2U
15	789.19	0	AG
55	798.42	0	AU
30	798.64	0	B2G
97	816.87	110.3913	B3U
21	817.82	0	B1G
14	824.95	0	AG
73	872.79	13.6407	B1U
29	921.68	0	B2G
54	925.86	0	AU
90	927.5	0.7088	B2U
72	951.93	8.5013	B1U
96	955.7	19.6728	B3U

28	960.66	0	B2G
47	970.82	0	B3G
53	986.86	0	AU
20	994.14	0	B1G
27	1013.07	0	B2G
95	1031.7	61.1008	B3U
89	1038.34	34.9484	B2U
13	1053.27	0	AG
19	1074.71	0	B1G
94	1075.12	6.898	B3U
52	1094.27	0	AU
26	1094.28	0	B2G
88	1105.38	1052.1347	B2U
71	1191.76	17.2852	B1U
87	1191.88	4916.5021	B2U
46	1207.22	0	B3G
12	1223.07	0	AG
45	1225	0	B3G
70	1243.9	56.3155	B1U
11	1255.64	0	AG
86	1261	564.7114	B2U
44	1308.43	0	B3G
69	1345.97	7.2323	B1U
43	1347.03	0	B3G
10	1353.41	0	AG
42	1369.12	0	B3G
68	1377.59	32.7413	B1U
85	1391.16	1760.1435	B2U
84	1398.18	478.7116	B2U
67	1428.58	20.8458	B1U
9	1453.92	0	AG
8	1462.27	0	AG
83	1468.79	3.4564	B2U
41	1494.16	0	B3G
66	1537.13	16.1403	B1U
82	1548.67	17.4605	B2U
40	1566.71	0	B3G
7	1566.97	0	AG
81	1590.19	284.9097	B2U
6	1628.76	0	AG
65	1632.89	192.4584	B1U
39	1642.64	0	B3G
80	1658.22	182.7294	B2U
5	1677.24	0	AG
64	1739.13	4.8394	B1U
38	1748.99	0	B3G

63	3237.83	4.3779	B1U
37	3238.14	0	B3G
4	3242.28	0	AG
79	3242.61	0.5393	B2U
62	3243.24	0.6257	B1U
36	3244.79	0	B3G
3	3245.7	0	AG
78	3247.03	10.1596	B2U
61	3248.05	12.6134	B1U
2	3251.92	0	AG
35	3256.28	0	B3G
60	3256.73	14.3634	B1U
77	3265.83	32.7997	B2U
1	3265.99	0	AG
	**TOTAL INT**	10100.78	

	**Total INT AU**	0	0	%
ip	**Total INT B1U**	491.071	4.861715431	%
ip	**Total INT B2U**	9345.6773	92.52434687	%
oop	**Total INT B3U**	264.028	2.613937703	%
	**Total INT**	10100.7763	100	%

**INT B3U / (INT B2U + INT B1U)**	0.026840984
**INT B3U / INT B2U**	0.02825135
**INT CHstr /INT CHoop**	0.285874983

Supplementary Table 10 | PENTACENE$^+$ (B/3-611G)**

mode	0.97*freq	INT	SYM
102	34.87	0.6487	B3U
59	63.38	0	AU
25	91.31	0	B1G
76	109.98	0.6553	B1U
34	135.58	0	B2G
101	171.59	1.5284	B3U
58	221.38	0	AU
51	222.81	0	B3G
18	240.18	0	AG
24	261.14	0	B1G
33	325.22	0	B2G
75	340.2	0.15	B1U
100	351.32	0.0442	B3U
99	412.63	29.9754	B3U
50	419.02	0	B3G
23	419.82	0	B1G
22	432.19	0	B1G
98	440.87	9.5763	B3U
93	449.01	0.0188	B2U
57	471.59	0	AU
49	472.5	0	B3G
32	497.82	0	B2G
74	536.72	1.3037	B1U
17	568.4	0	AG
92	589.73	0.1251	B2U
16	593.23	0	AG
56	642.66	0	AU
48	670.28	0	B3G
91	677.43	2.9029	B2U
31	680.8	0	B2G
15	686.89	0	AG
97	704.05	80.0636	B3U
55	704.1	0	AU
21	705.08	0	B1G
30	707.57	0	B2G
14	724.31	0	AG
73	776.48	0.0548	B1U
29	790.74	0	B2G
54	800.24	0	AU
96	801.19	12.2163	B3U
90	815.11	0.104	B2U

28	821.81	0	B2G
20	837.24	0	B1G
53	844.13	0	AU
72	850.48	0.98	B1U
27	861.45	0	B2G
95	866.7	59.5915	B3U
47	867.07	0	B3G
19	902.59	0	B1G
94	903.1	6.5372	B3U
52	928.87	0	AU
26	928.93	0	B2G
89	943.88	0.0003	B2U
13	944.94	0	AG
71	1048.64	1.3216	B1U
46	1064.1	0	B3G
88	1089.2	77.0963	B2U
45	1109.88	0	B3G
12	1115.5	0	AG
87	1115.88	69.5869	B2U
11	1120.38	0	AG
70	1122.2	0.4819	B1U
44	1144.31	0	B3G
86	1149.1	170.5443	B2U
43	1196.3	0	B3G
10	1196.74	0	AG
69	1204.11	7.5777	B1U
68	1208.85	27.7024	B1U
42	1219.33	0	B3G
85	1250.17	561.3502	B2U
9	1250.99	0	AG
67	1254.71	1.6893	B1U
84	1258.2	3.8555	B2U
8	1275.84	0	AG
83	1291.54	51.2222	B2U
41	1324.13	0	B3G
66	1353.56	4.1878	B1U
82	1369.94	7.1857	B2U
7	1383.01	0	AG
81	1392.06	355.8449	B2U
6	1394.66	0	AG
40	1410.34	0	B3G
80	1411.75	124.8564	B2U
5	1415.7	0	AG
39	1419.17	0	B3G
65	1454.82	0.2179	B1U
64	1472.86	25.5996	B1U

38	1481.12	0	B3G
63	2909.92	0.4976	B1U
4	2911.69	0	AG
37	2912.28	0	B3G
79	2914.1	1.4405	B2U
62	2915.19	7.5211	B1U
36	2917.31	0	B3G
3	2917.38	0	AG
61	2917.68	20.1091	B1U
78	2921.03	3.7789	B2U
2	2921.51	0	AG
35	2931.98	0	B3G
60	2932.01	34.0797	B1U
77	2942.38	75.8444	B2U
1	2942.54	0	AG
	**Total INT**	1840.068	

	**Total INT AU**	0	0	%
ip	**Total INT B1U**	134.1295	7.289375765	%
ip	**Total INT B2U**	1505.7573	81.83159387	%
oop	**Total INT B3U**	200.1816	10.87903037	%
	**Total INT**	1840.0684	100	%

**INT B3U / (INT B2U + INT B1U)**	0.122070377
**INT B3U / INT B2U**	0.132944134
**INT CHstr /INT CHoop**	0.715706638

Supplementary Table 11 | PENTACENE^{+} (BLYP/3-611G)**

mode	0.97*freq	INT	SYM
102	35.7	0.6984	B3U
59	66.03	0	AU
25	93.03	0	B1G
76	112.74	0.5681	B1U
34	139.71	0	B2G
101	174.73	1.6385	B3U
51	226.51	0	B3G
58	227.33	0	AU
18	247.79	0	AG
24	266.17	0	B1G
33	333.68	0	B2G
75	344.32	0.1813	B1U
100	361	0.0318	B3U
99	422.25	33.2693	B3U
50	425.33	0	B3G
23	430.38	0	B1G
22	441.61	0	B1G
98	451.22	8.9552	B3U
93	461.53	0.0627	B2U
49	479.15	0	B3G
57	481.12	0	AU
32	509.58	0	B2G
74	549.53	0.9127	B1U
17	580.25	0	AG
92	600.87	0.1701	B2U
16	603.73	0	AG
56	658.83	0	AU
48	687.63	0	B3G
31	697.68	0	B2G
91	705.27	2.3399	B2U
15	714.66	0	AG
97	718.15	84.2595	B3U
21	719.05	0	B1G
55	720.45	0	AU
30	723.68	0	B2G
14	753.42	0	AG
73	795.23	0.0416	B1U
29	808.1	0	B2G
54	817.8	0	AU
96	820.71	12.6086	B3U
90	840.83	0.2892	B2U

28	841.85	0	B2G
20	858.47	0	B1G
53	865.98	0	AU
72	868.3	0.8518	B1U
47	883.39	0	B3G
27	884.49	0	B2G
95	889.95	61.5156	B3U
19	925.72	0	B1G
94	926.36	8.3561	B3U
52	954.13	0	AU
26	954.19	0	B2G
89	977.61	0.0661	B2U
13	978.43	0	AG
71	1079.09	2.2534	B1U
46	1091.15	0	B3G
88	1121.04	46.021	B2U
12	1132.85	0	AG
45	1134.44	0	B3G
87	1135.47	106.5433	B2U
70	1147.47	0.7748	B1U
11	1151.33	0	AG
44	1183.56	0	B3G
86	1187.24	51.9326	B2U
43	1225.53	0	B3G
69	1227.53	2.0664	B1U
42	1238.44	0	B3G
68	1244.4	32.8753	B1U
10	1248.37	0	AG
67	1290.2	4.989	B1U
85	1315.04	452.2055	B2U
9	1318.17	0	AG
84	1325.15	0.8011	B2U
8	1336.14	0	AG
83	1341.48	143.7898	B2U
41	1349.4	0	B3G
66	1391.76	5.3725	B1U
82	1392.91	20.4581	B2U
7	1415.37	0	AG
81	1435.39	268.6711	B2U
6	1446.53	0	AG
80	1462.56	224.5662	B2U
40	1466.12	0	B3G
5	1468.1	0	AG
39	1469.44	0	B3G
65	1513.98	0.7532	B1U
64	1526.7	28.5365	B1U

38	1536.22	0	B3G
63	2999.49	0.3568	B1U
37	3001.23	0	B3G
4	3001.33	0	AG
79	3002.95	0.7439	B2U
62	3003.37	8.0135	B1U
3	3005.27	0	AG
36	3007.82	0	B3G
61	3007.82	3.7106	B1U
78	3010.99	2.7398	B2U
2	3011.14	0	AG
35	3022.25	0	B3G
60	3022.26	13.2317	B1U
77	3032.1	26.6579	B2U
1	3032.16	0	AG
	**Total INT**	1664.880	

	**Total INT AU**	0	0	%
ip	**Total INT B1U**	105.4892	6.336142444	%
ip	**Total INT B2U**	1348.0583	80.97027384	%
oop	**Total INT B3U**	211.333	12.69358371	%
	**Total INT**	1664.8805	100	%

**INT B3U / (INT B2U + INT B1U)**	0.145391189
**INT B3U / INT B2U**	0.156768442
**INT CHstr /INT CHoop**	0.262401991

Supplementary Table 12 | PICENE (B3LYP/3-611G)**

mode	0.97*freq	INT	SYM
68	38.11	0.2505	B1
52	45.46	0	A2
67	100.07	0.0479	B1
51	105.83	0	A2
35	137.42	0.3395	A1
50	178.55	0	A2
66	204.21	2.4953	B1
65	247.37	6.1558	B1
34	252.91	0.0635	A1
102	274.28	0.0066	B2
49	304.22	0	A2
48	380.81	0	A2
64	409.07	9.0444	B1
33	414.47	0.3346	A1
47	459.66	0	A2
101	462.52	1.0106	B2
100	473.51	11.6932	B2
63	492.45	0.0121	B1
32	511.83	0.0008	A1
99	513.2	6.4836	B2
62	518.68	8.0357	B1
46	542.35	0	A2
98	544.83	4.0739	B2
45	576.7	0	A2
31	578.28	0.3093	A1
30	635.99	0.021	A1
61	639.32	0.2002	B1
97	666.76	15.3987	B2
44	694.28	0	A2
29	720.78	0.1398	A1
60	725.98	13.0544	B1
43	739.31	0	A2
59	742.11	74.5891	B1
42	775.06	0	A2
96	776.9	0.8242	B2
58	792.65	79.7959	B1
57	805.43	20.459	B1
41	813.32	0	A2
95	842.97	12.6727	B2
40	847.48	0	A2

56	850.11	8.9156	B1
28	850.46	0.5256	A1
94	866.61	0.1543	B2
27	898.08	0.0158	A1
39	907.83	0	A2
38	922.08	0	A2
55	926.26	1.8111	B1
37	937.69	0	A2
54	944.9	0.5118	B1
53	962.79	0.1168	B1
36	963.16	0	A2
93	1021.98	8.127	B2
26	1025.65	1.0877	A1
92	1037.57	1.8045	B2
25	1063.85	0.5069	A1
91	1121.48	2.8472	B2
24	1133.53	1.4621	A1
23	1141.79	0.0063	A1
90	1145.68	0.1906	B2
89	1156.95	1.9154	B2
22	1174.64	2.0283	A1
21	1181.75	0.1135	A1
88	1203.93	0.5191	B2
87	1218.51	2.9838	B2
20	1228.73	0.0663	A1
86	1248.78	0.6067	B2
19	1252.2	14.2745	A1
18	1258.99	8.281	A1
85	1308.39	0.2519	B2
17	1322.51	0.9531	A1
16	1338.85	0.3727	A1
84	1339.58	0.0055	B2
15	1358.42	0.2284	A1
83	1380.81	0.3542	B2
82	1414.02	2.7784	B2
14	1419.69	8.1463	A1
13	1425.26	0.3873	A1
81	1438.3	11.4639	B2
80	1468.2	14.8549	B2
12	1498.77	0.9105	A1
11	1509.29	0.187	A1
79	1521.1	3.2141	B2
78	1560.59	0.3088	B2
10	1578.57	3.7219	A1
77	1595.84	1.3555	B2
9	1602.95	3.3108	A1

8	1606.87	0.0541	A1
76	1612.51	1.3397	B2
75	3064.43	0.6484	B2
7	3064.57	0.3424	A1
74	3069.04	4.7739	B2
6	3070.09	11.9216	A1
73	3074.99	0.4529	B2
5	3075.19	37.0059	A1
72	3089.05	61.8627	B2
4	3090.1	0.7126	A1
71	3094.67	15.7993	B2
3	3102.95	5.6987	A1
70	3107.5	0.1249	B2
69	3111.73	2.8181	B2
2	3120.14	30.7646	A1
1	3126	19.853	A1
	**Total INT**	573.362	

ip	**Total INT A1**	154.1474	26.8848208	%
	**Total INT A2**	0	0	%
oop	**Total INT B1**	225.4956	39.32864776	%
ip	**Total INT B2**	193.7192	33.78653145	%
	Total INT	573.3622	100	%

**INT B1/ (INT B2 + INT A1)**	0.648224348
**INT B1 / INT B2**	1.164033302
**INT CHstr /INT CHoop**	0.854912468

Supplementary Table 13 | PICENE$^+$ (B3LYP/3-611G)**

mode	0.97*freq	INT	SYM
52	32.25	0	A2
68	38.74	0.3882	B1
67	90.03	0.2066	B1
51	105.27	0	A2
35	135.11	0.0307	A1
50	166.82	0	A2
66	199.39	4.6229	B1
65	240.44	8.7522	B1
34	250.76	0.0203	A1
102	267.97	22.6986	B2
49	299.86	0	A2
48	366.08	0	A2
64	398.25	7.3019	B1
33	411.38	0.4717	A1
47	438.08	0	A2
101	451.44	0.0066	B2
100	460.4	109.6179	B2
63	477.45	5.0595	B1
62	492.53	3.2591	B1
46	503.94	0	A2
32	508.01	0.222	A1
99	509.92	0.1354	B2
45	531.45	0	A2
98	540.23	38.0827	B2
31	573.87	0.6632	A1
30	632.95	0.0074	A1
61	642.06	3.2531	B1
97	657.32	36.0578	B2
44	697.36	0	A2
29	719.45	0.1477	A1
60	733.68	4.8405	B1
59	746.57	89.4721	B1
43	746.81	0	A2
42	759.96	0	A2
96	775.62	19.8569	B2
58	817.6	63.5313	B1
41	822.89	0	A2
57	831.6	21.2668	B1
95	841.71	60.8205	B2
28	848.92	0.2392	A1
94	860.27	20.4178	B2

40	860.77	0	A2
56	864.09	9.4879	B1
27	893.44	0.4453	A1
39	939.37	0	A2
38	947.09	0	A2
55	949.51	1.1561	B1
37	961.61	0	A2
54	965.84	1.3424	B1
53	989.87	0.0465	B1
36	990.22	0	A2
93	1019.1	0.2144	B2
26	1027.92	0.1781	A1
92	1039.74	0.223	B2
25	1070.93	0.2087	A1
91	1118.85	145.3226	B2
24	1131.2	0.647	A1
90	1133.56	189.5533	B2
23	1157.13	2.6399	A1
89	1157.49	164.5804	B2
22	1176.4	0.0359	A1
21	1211.51	32.1451	A1
88	1211.59	18.501	B2
87	1231.77	497.1644	B2
20	1242	7.8422	A1
19	1247.52	0.9747	A1
18	1261.13	0.879	A1
86	1261.83	0.8258	B2
85	1305.49	8.6457	B2
17	1314.28	1.0725	A1
84	1317.54	1407.3923	B2
16	1337.03	0.0233	A1
15	1354.31	1.7409	A1
83	1375.11	352.6698	B2
82	1414.99	8.5616	B2
14	1418.62	1.7292	A1
81	1420.26	0.6314	B2
13	1427.37	5.371	A1
12	1491.06	9.7038	A1
80	1491.72	120.3795	B2
79	1501.5	53.6698	B2
11	1513.39	1.3817	A1
78	1528.01	1066.5946	B2
10	1531.63	28.5595	A1
77	1537.23	272.5297	B2
9	1567.44	45.9853	A1
76	1576.52	250.378	B2

8	1589.37	24.1601	A1
75	3085.32	0.0685	B2
7	3085.71	0.1032	A1
74	3088.16	2.0456	B2
6	3088.62	1.5622	A1
73	3093.42	0.276	B2
5	3093.67	2.6928	A1
72	3105.06	5.8945	B2
4	3107.91	1.4003	A1
71	3110.41	15.4715	B2
3	3113.47	1.4402	A1
70	3119.33	2.0567	B2
69	3120.38	8.4914	B2
2	3128.13	4.1465	A1
1	3135.16	2.7479	A1
	**Total INT**	5305.441	

ip	**Total INT A1**	181.6185	3.423249636	%
	**Total INT A2**	0	0	%
oop	**Total INT B1**	223.9871	4.221837305	%
ip	**Total INT B2**	4899.8357	92.35491306	%
	Total INT	5305.4413	100	%

**INT B1/ (INT B2 + INT A1)**	0.044079331
**INT B1 / INT B2**	0.045713186
**INT CHstr /INT CHoop**	0.216071818

Supplementary Table 14 | PICENE$^+$ (ROHF/6-311G)**

mode	0.97*freq	INT	SYM
52	27.63	0	A2
68	42.87	0.1473	B1
67	98.03	0.5471	B1
51	114.43	0	A2
35	141.82	0.0017	A1
50	183.01	0	A2
66	216.31	5.6517	B1
102	227.31	2246.2069	B2
65	262.51	11.4331	B1
34	265.27	0.0232	A1
49	329.34	0	A2
101	396.14	2931.6461	B2
48	401.35	0	A2
64	436.9	5.6064	B1
33	437.25	1.1477	A1
47	473.32	0	A2
100	486.21	41.4056	B2
63	524.97	7.7594	B1
99	529.39	157.6648	B2
62	534.72	7.7008	B1
32	536.97	0.2483	A1
46	550.57	0	A2
98	563.98	322.4801	B2
45	578.26	0	A2
31	612.35	2.7286	A1
30	673.11	0.9495	A1
97	691.12	620.3531	B2
61	695.69	1.618	B1
44	748.78	0	A2
29	763.19	0.0004	A1
60	791.03	4.4875	B1
96	794.83	2920.095	B2
43	809.42	0	A2
59	822.98	112.5247	B1
42	823.35	0	A2
95	851.17	1447.2045	B2
41	896.18	0	A2
58	898.39	68.2982	B1
94	904.69	4.0859	B2
28	905.39	1.7676	A1
57	921.42	25.7857	B1

27	941.95	0.2501	A1
40	942.78	0	A2
56	945.34	9.0897	B1
93	1001.41	2929.9685	B2
39	1042.26	0	A2
38	1057.77	0	A2
55	1060.03	1.543	B1
37	1074.61	0	A2
54	1080.95	0.7894	B1
26	1082.6	9.7324	A1
53	1094.94	0.1157	B1
36	1095.19	0	A2
92	1095.54	4116.7759	B2
91	1109.63	955.8883	B2
25	1133.8	27.4179	A1
90	1162.29	1791.6842	B2
24	1167.78	43.1508	A1
23	1205.4	2.5093	A1
89	1219.77	157.2999	B2
22	1259.13	5.3779	A1
88	1263.59	184.3282	B2
21	1266.08	11.2097	A1
87	1282.14	2431.1312	B2
20	1318.12	0.2867	A1
86	1327.32	758.1742	B2
19	1330.82	0.1807	A1
18	1345.83	6.0534	A1
85	1354.01	10.7715	B2
17	1376.44	11.2245	A1
84	1393.57	232.9323	B2
16	1410.84	0.0007	A1
83	1416.92	130.0598	B2
15	1436.5	32.5554	A1
14	1511.49	5.0108	A1
82	1522.75	4.9448	B2
13	1534.17	5.8301	A1
81	1535.12	4.1893	B2
80	1585.44	999.8697	B2
12	1595.56	18.7925	A1
79	1614.63	10.7731	B2
11	1630.59	23.1447	A1
78	1634.56	392.5295	B2
10	1672.44	75.8489	A1
77	1676.6	185.9006	B2
9	1716.8	84.3809	A1
76	1731.68	155.9749	B2

8	1738.62	16.9591	A1
75	3240.41	0.0072	B2
7	3240.48	0.0043	A1
74	3246.62	7.1549	B2
6	3247.01	0.0799	A1
73	3249.67	8.9119	B2
5	3249.98	12.1179	A1
72	3265.17	24.8926	B2
4	3265.61	1.0694	A1
71	3271.46	3.0329	B2
3	3275.13	3.7615	A1
70	3292.01	2.732	B2
69	3295.42	3.479	B2
2	3305.88	5.0954	A1
1	3321.61	1.8005	A1
	**Total INT**	26868.358	

ip	**Total INT A1**	410.7124	1.528609945	%
	**Total INT A2**	0	0	%
oop	**Total INT B1**	263.0977	0.979210174	%
ip	**Total INT B2**	26194.5484	97.49217988	%
	**Total INT**	26868.3585	100	%

**INT B1/ (INT B2 + INT A1)**	0.009888935
**INT B1 / INT B2**	0.010043987
**INT CHstr /INT CHoop**	0.281794178

Supplementary Table 15 | PICENE$^+$ (B/3-611G)**

mode	0.97*freq	INT	SYM
52	25.3	0	A2
68	33.74	0.3596	B1
67	78.74	0.1905	B1
51	96.44	0	A2
35	128.46	0.0494	A1
50	155.2	0	A2
66	187.68	3.7865	B1
65	227.62	7.368	B1
34	234.94	0.0188	A1
102	255.43	11.6182	B2
49	280.47	0	A2
48	342.8	0	A2
64	374.61	7.3096	B1
33	391.86	0.4013	A1
47	414.59	0	A2
101	426.79	0.0164	B2
100	440.95	50.9613	B2
63	451.19	3.9663	B1
62	465.92	2.3025	B1
46	476.62	0	A2
32	484.66	0.1533	A1
99	485.21	0.0302	B2
45	501.89	0	A2
98	515.07	15.6969	B2
31	544.15	0.4039	A1
30	597.47	0.0242	A1
61	605.2	2.9795	B1
97	621.56	24.3185	B2
44	659.8	0	A2
29	676.95	0.1955	A1
60	695.07	6.5925	B1
59	704.79	75.2391	B1
43	705.19	0	A2
42	721.45	0	A2
96	735.11	15.7678	B2
58	769.37	53.5431	B1
41	774.06	0	A2
57	779.58	24.5288	B1
95	795.28	26.858	B2
28	801.62	0.1246	A1
40	809.54	0	A2

56	813.07	8.105	B1
94	813.82	10.1822	B2
27	836.74	0.6788	A1
39	879.17	0	A2
38	884.78	0	A2
55	888.4	0.8357	B1
37	899.74	0	A2
54	902.96	1.3337	B1
53	925.08	0.043	B1
36	925.41	0	A2
93	954.32	0.512	B2
26	965.5	0.346	A1
92	981.88	0.0027	B2
25	1005.68	0.4858	A1
91	1062.33	30.0097	B2
24	1076.14	0.6884	A1
90	1084.43	75.5787	B2
89	1108.94	113.1186	B2
23	1109.59	8.5	A1
22	1109.87	0.0462	A1
21	1125.62	6.7563	A1
88	1143.66	45.3887	B2
87	1162.93	399.9722	B2
20	1166.9	5.5029	A1
19	1179.02	2.791	A1
18	1188.89	1.0997	A1
86	1192.51	44.2872	B2
17	1206.96	0.6305	A1
85	1213.92	164.6755	B2
84	1230.26	337.9196	B2
16	1233.09	2.1184	A1
15	1256.64	0.7678	A1
83	1284.82	110.8	B2
82	1326.26	85.559	B2
14	1334.96	0.2513	A1
81	1347.79	0.0151	B2
13	1351.52	4.6191	A1
80	1398.54	83.6147	B2
12	1402.31	13.1835	A1
79	1407.03	22.5334	B2
11	1418.25	4.2432	A1
10	1425.91	16.449	A1
78	1430.7	717.0743	B2
77	1437	20.6193	B2
9	1451.41	26.8729	A1
76	1461.8	147.9474	B2

8	1470.49	29.625	A1
75	2917.24	0.0951	B2
7	2917.58	0.2067	A1
74	2921.09	6.874	B2
6	2921.65	5.8076	A1
73	2926.23	0.2092	B2
5	2926.5	14.3087	A1
72	2938.94	19.9053	B2
4	2941.32	2.4446	A1
71	2943.93	51.359	B2
3	2948.13	5.1212	A1
70	2954.16	2.7605	B2
69	2956.28	14.5092	B2
2	2964.97	17.3728	A1
1	2970.64	11.5966	A1
	**Total INT**	3033.1583	

ip	**Total INT A1**	183.885	6.062492683	%
	**Total INT A2**	0	0	%
oop	**Total INT B1**	198.4834	6.543786389	%
ip	**Total INT B2**	2650.7899	87.39372093	%
	Total INT	3033.1583	100	%

**INT B1/ (INT B2 + INT A1)**	0.070019811
**INT B1 / INT B2**	0.074877077
**INT CHstr /INT CHoop**	0.768681411

Supplementary Table 16 | PICENE$^+$ (BLYP/3-611G**)

mode	0.97*freq	INT	SYM
52	31.83	0	A2
68	36.37	0.4459	B1
67	86.15	0.17	B1
51	100.96	0	A2
35	131.82	0.0495	A1
50	160.88	0	A2
66	192.3	4.0762	B1
65	232.23	7.7909	B1
34	243.54	0.0229	A1
102	261.97	10.0945	B2
49	288.19	0	A2
48	352.02	0	A2
64	383.21	7.3721	B1
33	400.91	0.3594	A1
47	423.93	0	A2
101	439.15	0.1054	B2
100	452.43	42.3346	B2
63	460.73	4.0836	B1
62	475.77	2.6452	B1
46	486.65	0	A2
32	495.43	0.1397	A1
99	497.48	0.8108	B2
45	513.47	0	A2
98	526.62	12.1226	B2
31	557.48	0.405	A1
30	614.71	0.0456	A1
61	619.31	3.4028	B1
97	638.62	20.6706	B2
44	675.09	0	A2
29	698.21	0.1794	A1
60	710.91	8.5912	B1
59	719.26	77.6322	B1
43	719.83	0	A2
42	737.68	0	A2
96	754.44	12.6784	B2
58	786.95	59.8346	B1
41	793.1	0	A2
57	798.42	21.0162	B1
95	818.12	21.7485	B2
28	824.44	0.1007	A1
40	829.45	0	A2
56	833.07	8.8385	B1

94	837.16	9.9188	B2
27	866.74	0.4859	A1
39	900.71	0	A2
38	907.59	0	A2
55	910.82	1.0657	B1
37	922.92	0	A2
54	926.47	1.456	B1
53	950.54	0.0401	B1
36	950.89	0	A2
93	988.9	0.9075	B2
26	997.32	0.1388	A1
92	1008.74	0.0007	B2
25	1037.68	0.1626	A1
91	1089.63	19.0185	B2
24	1101.11	0.4111	A1
90	1106.74	69.37	B2
23	1131.29	0.9034	A1
89	1131.63	69.0581	B2
22	1141.6	0.0469	A1
21	1175.58	21.1497	A1
88	1180.33	74.5846	B2
87	1202.11	155.7428	B2
20	1208.29	12.2035	A1
19	1208.84	0.0017	A1
18	1222.09	0.8693	A1
86	1225.03	1.0093	B2
85	1262.78	55.95	B2
17	1269.71	0.9249	A1
84	1285.54	604.5836	B2
16	1287.96	0.1987	A1
15	1312.51	0.96	A1
83	1334.85	99.0736	B2
82	1370.93	67.4885	B2
14	1374.29	0.8378	A1
81	1376.66	0.1347	B2
13	1383.94	6.0314	A1
80	1440.14	58.5038	B2
12	1444.73	6.8394	A1
79	1448.58	2.305	B2
11	1460.95	0.5168	A1
10	1473.43	24.5549	A1
78	1480.43	79.7945	B2
77	1483.94	674.9406	B2
9	1506.23	31.7632	A1
76	1514.89	206.4024	B2
8	1526.01	25.4176	A1

75	3007.47	0.0126	B2
7	3007.96	0.254	A1
74	3010.71	2.999	B2
6	3011.08	3.137	A1
73	3016.3	0.0201	B2
5	3016.64	3.3424	A1
72	3026.15	3.4143	B2
4	3030.11	2.7129	A1
71	3032.83	18.4661	B2
3	3035.63	1.2579	A1
70	3038.93	1.4151	B2
69	3039.77	11.2973	B2
2	3046.6	7.4634	A1
1	3051.74	5.1324	A1
	**Total INT**	2774.4579	

ip	**Total INT A1**	159.0198	5.731562912	%
	**Total INT A2**	0	0	%
oop	**Total INT B1**	208.4612	7.513583104	%
ip	**Total INT B2**	2406.9769	86.75485398	%
	Total INT	2774.4579	100	%

**INT B1/ (INT B2 + INT A1)**	0.081239855
**INT B1 / INT B2**	0.086607063
**INT CHstr /INT CHoop**	0.292258224

Supplementary Table 17 | All-in mode contributions (%) to IR activity

	ROHF	*B*	*BLYP*	*B3LYP*	*Modes*
**Picene$^+$**					
$35A_1$	1.53	6.06	5.73	3.42 (27)	$\nu_1, \nu_2 \ldots \nu_{35}$
$16B_1$	0.98	6.54	7.51	4.22 (39)	$\nu_{53}, \nu_{54} \ldots \nu_{68}$
$34B_2$	97.49	87.39	86.76	92.36 (34)	$\nu_{69}, \nu_{70} \ldots \nu_{102}$
**Pentacene$^+$**					
$17B_{1u}$	4.86	7.29	6.33	3.61 (32)	$\nu_{60}, \nu_{61} \ldots \nu_{76}$
$17B_{2u}$	92.53	81.83	80.97	88.16 (27)	$\nu_{77}, \nu_{78} \ldots \nu_{93}$
$9B_{3u}$	2.61	10.88	12.7	8.23 (41)	$\nu_{94}, \nu_{95} \ldots \nu_{102}$

IR-active modes grouped by symmetry of picene$^+$ (C_{2v}) and pentacene$^+$ (D_{2h}) according to ROHF, B, BLYP and B3LYP quantum-chemical methods. The contributions in parenthesis belong to neutral state. The basis set used is 6-311G**.

Supplementary Table 18 | C_2H_2 (1) and C_4H_4 (2) loss ion signals of picene$^+$ action bands recorded without FEL power-level attenuation (0 dB; B0B1)

	(1) *m/z* 252 -- 0 dB		(2) *m/z* 226 -- 0 dB			
band	$\tilde{\nu}_{exp}$ (cm^{-1})	S_{ion} (arb. u.)	$\tilde{\nu}_{exp}$ (cm^{-1})	S_{ion} (arb. u.)	$\Delta\tilde{\nu}_{exp}^{(1)-(2)}$	$\lvert\Delta\tilde{\nu}_{exp}^{(1)-(2)}\rvert$
a_r	-	-	-	-	-	-
a	1505	0.000533006	1484	0.00031194	0.35	0.35
b_r	1337	0.0005077	1319	0.000322702	0.34	0.34
b	1225	0.00074627	1201	0.000454415	0.49	0.49
b_l	1125	0.00070342	1123	0.00039893	0.04	0.04
c	1057	0.00059413	1034	0.000348423	0.55	0.55
d	962	0.0005322	953	0.000308214	0.23	0.23
e	820	0.00061566	826	0.00030976	-0.18	0.18
f	736	0.00080505	731	0.000339689	0.17	0.17
					avg	0.30
					sdev	0.16

Supplementary Table 19 | C_2H_2 (1) and C_4H_4 (2) loss ion signals of picene$^+$ action bands recorded with FEL power-level attenuation (3 dB; B2B3)

	(1) *m/z* 252 -- 3 dB		(2) *m/z* 226 -- 3 dB			
band	$\tilde{v}_{exp}$ (cm^{-1})	S_{ion} (arb. u.)	$\tilde{v}_{exp}$ (cm^{-1})	S_{ion} (arb. u.)	$\Delta\tilde{v}_{exp}^{(1)\text{-}(2)}$	$\lvert\Delta\tilde{v}_{exp}^{(1)\text{-}(2)}\rvert$
a_r	1543	0.00022688	1540	0.00012263	0.05	0.05
a	1468	0.00037044	1456	0.000171933	0.21	0.21
b_r	1344	0.00064723	1331	0.0003022	0.24	0.24
b	1282	0.00087385	1260	0.000390832	0.43	0.43
b_l	1211	0.00086801	1191	0.000326252	0.42	0.42
c	1132	0.00074201	1139	0.000305964	-0.15	0.15
d	992	0.00040121	1004	0.000166622	-0.30	0.30
e	822	0.00063338	824	0.000191013	-0.06	0.06
f	741	0.00069057	744	0.000210812	-0.10	0.10
					avg	0.22
					sdev	0.13

Supplementary Table 20 | Percentage differences between band frequencies and product ion signals, and channel ion signal ratios at 0 and 3 dB FEL power levels. Signals increments are positive (+) and decrements are negative (-).

	(1) *m/z* 252		(2) *m/z* 226		(1) *m/z* 252	(2) *m/z* 226	*m/z* 252 : *m/z* 226	
band	$\Delta\tilde{v}_{exp3dB\text{-}0dB}^{(1)}$	$\lvert\Delta\tilde{v}_{exp3dB\text{-}0dB}^{(1)}\rvert$	$\Delta\tilde{v}_{exp3dB\text{-}0dB}^{(2)}$	$\lvert\Delta\tilde{v}_{exp3dB\text{-}0dB}^{(2)}\rvert$	$\Delta S_{0dB\text{-}3dB}^{(1)}$	$\Delta S_{0dB\text{-}3dB}^{(2)}$	0 dB	3 dB
a_r	-	-	-	-	-	-	-	1.85
a	-0.62	0.62	-0.48	0.48	-30	-45	1.71	2.15
b_r	0.13	0.13	0.23	0.23	27	-6	1.57	2.14
b	1.14	1.14	1.20	1.20	17	-14	1.64	2.24
b_l	1.84	1.84	1.47	1.47	23	-18	1.76	2.66
c	1.71	1.71	2.42	2.42	25	-12	1.71	2.43
d	0.77	0.77	1.30	1.30	-25	-46	1.73	2.41
e	0.06	0.06	-0.06	0.06	3	-38	1.99	3.32
f	0.17	0.17	0.44	0.44	-14	-38	2.37	3.28
	avg	0.81	*avg*	0.95		*avg*	1.81	2.58
	sdev	0.66	*sdev*	0.74		*sdev*	0.24	0.44

Supplementary Table 21 | Characterization of absolute dissociation yield (β^*) action bands of picene$^+$ (B0B1 data set) recorded without FEL power-level attenuation

IR multiple-photon action			Deconvoluted band fit components							
band	$\tilde{\nu}_{exp}$	β^*	*Peak*	$\tilde{\nu}_c$	ε	*w*	ε	*A*	ε	*h*
a_r	1537	0.008175	1	1541	*9.47*	43	*43.48*	0.18	*0.30*	0.0026
a	1492	0.010312	2	1494	*9.71*	90	*26.00*	1.08	*0.49*	0.0076
b_r	1312	0.011526	3	1324	*14.79*	159	*54.30*	1.58	*1.00*	0.0064
b	1207	0.016211	4	1208	*6.33*	148	*45.28*	2.79	*1.34*	0.0120
b_l	1126	0.014511	5	1122	*6.33*	70	*40.23*	0.52	*0.56*	0.0048
c	1044	0.01412	6	1044	*6.15*	108	*41.18*	1.33	*0.81*	0.0079
d	966	0.012023	7	961	*9.61*	121	*26.83*	1.35	*0.61*	0.0071
e	818	0.01247	8	818	*1.45*	82	*6.55*	1.27	*0.11*	0.0098
f	735	0.015548	9	734	*0.53*	33	*1.86*	0.64	*0.03*	0.0124

Definition of column headings in Table 2. Statistics: Reduced Chi-Sqr 8.85E-07, Residual Sum of Squares 1.76E-04, R-Square(COD) 0.94613, Adj. R-Square 0.9391.

Supplementary Table 22 | Characterization of absolute dissociation yield (β^*) action bands of picene$^+$ (B2B3 data set) recorded with FEL power-level attenuation (3 dB)

IR multiple-photon action			Deconvoluted band fit components							
band	$\tilde{\nu}_{exp}$	β^*	*Peak*	$\tilde{\nu}_c$	ε	*w*	ε	*A*	ε	*h*
a_r	1518	0.00601	1	1521	*4.53*	39	*14.96*	0.19	*0.08*	0.0031
a	1472	0.007436	2	1473	*3.94*	48	*14.40*	0.32	*0.11*	0.0042
b_r	1290	0.01343	3	1291	*11.45*	183	*20.78*	3.02	*0.70*	0.0105
b	1219	0.01527	4	1211	*4.85*	95	*31.93*	1.00	*0.63*	0.0067
b_l	1131	0.01369	5	1129	*3.34*	86	*17.49*	1.01	*0.32*	0.0075
c	1028	0.008556	6	1024	*10.47*	138	*19.48*	1.15	*0.21*	0.0053
d	986	0.007722	7	986	*5.14*	30	*25.67*	0.07	*0.08*	0.0014
e	824	0.007069	8	823	*1.56*	73	*6.28*	0.63	*0.05*	0.0055
f	741	0.007715	9	741	*0.73*	27	*2.45*	0.25	*0.02*	0.0058

Definition of column headings in Table 2. Statistics: Reduced Chi-Sqr 9.40E-07, Residual Sum of Squares 3.91E-04, R-Square (COD) 0.94544, Adj. R-Square 0.94203.

Supplementary Table 23 | Characterization of absolute dissociation yield (β^*) action bands of pentacene$^+$ (D6D7 data set) recorded at power values between B0B1 and B2B3 power values

IR multiple-photon action			Deconvoluted band fit components							
band	$\tilde{v}_{exp}$	β^*	*Peak*	$\tilde{v}_c$	ε	w	ε	A	ε	h
a_r	1524	0.0041651	1	1528	*5.36*	54	*22.26*	0.18	*0.09*	0.0021
a	1437	0.0075038	2	1443	*4.44*	102	*24.08*	0.89	*0.29*	0.0056
b_r	-	-	3	1351	*6.79*	89	*38.99*	0.57	*0.39*	0.0041
b	1313	0.0125	4	1308	*0.00*	91	*7.48*	1.29	*0.21*	0.0091
c_r	1196	0.0120123	5	1201	*2.36*	50	*6.93*	0.63	*0.13*	0.0081
c	1173	0.0141172	6	1170	*1.06*	27	*5.48*	0.33	*0.10*	0.0077
c_l	1139	0.0106807	7	1136	*1.33*	39	*4.26*	0.46	*0.06*	0.0074
d	1076	0.0048443	8	1074	*1.40*	25	*4.86*	0.12	*0.02*	0.0030
e_r	970	0.004284	9	972	*2.60*	43	*9.34*	0.15	*0.03*	0.0022
e	905	0.0074332	10	905	1.23	74	4.42	0.81	0.06	0.0070
e_l	-	-	-	-	-	-	-	-	-	-
f	739	0.008938	11	738	0.41	39	1.68	0.53	0.02	0.0087

Definition of column headings in Table 2. Statistics: Reduced Chi-Sqr 8.68E-07, Residual Sum of Squares 6.81E-04, R-Square (COD) 0.92799, Adj. R-Square 0.92505.

Supplementary Note 1

First-order mode perturbation expansion of Ψ_g The molecular electronic ground-state wavefunction Ψ_g at the equilibrium geometry written as a first-order expansion[1] of adiabatic one-electron state wavefunctions Ψ_ξ with respect to the nuclear displacement normal coordinate Q:

$$\Psi_g(r;Q) = \Psi_o(r,0) + \sum_i \frac{\langle \Psi_o(r,0)|(\partial H/\partial Q)_0|\Psi_i(r,0)\rangle Q}{E_i - E_o} \Psi_i(r,0) \tag{1}$$

where the derivatives are evaluated at the equilibrium position (Q_0) represented by the subscript 0. The wavefunctions Ψ_ξ (ξ=*o:* ground state; ξ=*i:* low-lying excited state) are anti-symmetric electronic configurations written as single Slater determinants of molecular orbital (MO) functions. The MOs are constructed from linear combinations of atomic orbitals (LCAOs). In this work the LCAOs are based on John Pople's split-valence triple-ζ basis set 6-311G with polarization functions *p* and *d* (see Methods).

Supplementary Reference

1. Marconi, G., Orlandi, G. & Malpezzi, L. Vibronic coupling and IR intensities. *Chem. Phys. Lett.* **61,** 545–547 (1979).
